# RatioNet: Ratio Prediction Network for Object Detection

**DOI:** 10.3390/s21051672

**Published:** 2021-03-01

**Authors:** Kuan Zhao, Boxuan Zhao, Lifang Wu, Meng Jian, Xu Liu

**Affiliations:** Department of Information, Beijing University of Technology, Beijing 100124, China; S201861077@emails.bjut.edu.cn (K.Z.); zhaoboxuan@emails.bjut.edu.cn (B.Z.); lfwu@bjut.edu.cn (L.W.); liuxu91@bjut.edu.cn (X.L.)

**Keywords:** object detection, bounding box regression, high-quality box detection

## Abstract

In object detection of remote sensing images, anchor-free detectors often suffer from false boxes and sample imbalance, due to the use of single oriented features and the key point-based boxing strategy. This paper presents a simple and effective anchor-free approach-RatioNet with less parameters and higher accuracy for sensing images, which assigns all points in ground-truth boxes as positive samples to alleviate the problem of sample imbalance. In dealing with false boxes from single oriented features, global features of objects is investigated to build a novel regression to predict boxes by predicting width and height of objects and corresponding ratios of l_ratio and t_ratio, which reflect the location of objects. Besides, we introduce ratio-center to assign different weights to pixels, which successfully preserves high-quality boxes and effectively facilitates the performance. On the MS-COCO test–dev set, the proposed RatioNet achieves 49.7% AP.

## 1. Introduction

Object detection has been promoted considerably with the advancement of deep learning, especially using convolutional neural networks (CNN). Filtering/convention plays a vital role in the field of object detection, improving the detection accuracy significantly by expressing neighborhood spatial connectivity patterns and extracting the semantic features of objects. CNNs can generate image representations that capture hierarchical patterns and gain global receptive fields. Specifically, object detection serves as a fundamental task in multiple visual domains, e.g., facial research [1,2], autonomous driving [3,4], image encryption and pose estimation [5,6]. The existing detectors can be classified as anchor-based detectors [7,8] and anchor-free detectors [9,10] evaluated on the benchmarks [11,12].

In anchor-based detectors, anchor boxes are the essential components, which are obliged to be designed precisely for the optimal regression. Despite tremendous improvements of detection with anchor boxes [13,14], the drawbacks come along seriously. First, extensive and dense anchor boxes are required for a high recall rate [15]. Meanwhile, computing intersection over union (IoU) between anchor boxes and ground-truth boxes brings a significant increment of computation and memory cost. Second, the scales and aspect ratios with an aborative design are sensitive to dataset, in other words, the hyper-parameters should be redesigned on a new detection task.

To address the above weakness of anchor-based methods, a great number of anchor-free approaches are proposed recently. Among them, keypoint-based detection approaches [16,17,18] have improved performance significantly by detecting object bounding boxes as corner, extreme or center points. Nevertheless, confined corner or center regions are applied for exact points detection, which results in a quantitative imbalance between positive and negative samples, further leading to hard training. On the other hand, a fully convolutional anchor-free detection network [19] achieves good accuracy and speed trade-off, where the distances from points to four boundaries are predicted separately. It mainly considers single oriented features in each channel. So, it is easy to regress false results even if only one oriented length is predicted by mistake because of ignoring the global features of the object.

We argue that all the pixels in the ground-truth box, including the corner and center pixels, provide a clue to the object. Intuitively representation capability of the pixels is variant. Therefore, it is essential to investigate how to utilize high-quality pixels. Moreover, compared to the four distances from a pixel to boundaries of the box, the width and height of the box reflect more global features of the object, since width or height indicates the distance from one side to the opposite side, which includes more information of the object. Additionally, the location of a pixel in the box could be accurately represented by the ratio relation between its distance to the left (top) boundary and the width (height) of the box.

Motivated by the above characteristic, we propose a simple and effective method RatioNet, which utilizes all pixels in ground-truth boxes as positive samples and thus alleviating the quantitative imbalance between positive and negative samples. The prediction of width and height is constructed as a subnet of RatioNet. What’s more, the ratio of the relative length (i.e., the distance from the pixel to the left (top) boudary) to the width (height), named as l_ratio (t_ratio) are utilized to locate the point’s location relative to the box, i.e., width and height help to determine the size of the box, while the points and corresponding ratios are responsible for the location of the box. On the other hand, in order to preserve more boxes of high-quality, we introduce a ratio-center by assigning weights to pixels according to the distance away from the center. The closer to the center is the pixel, the higher weight is assigned. We use the feature pyramid network to integrate the features of high levels and low levels for better object detection especially the small ones. In this way, accurate boxes get high scores while those low-quality boxes are eliminated through post-processing, e.g., NMS [20]. The proposed RatioNet is evaluated on MS-COCO [2], a challenging benchmark for large-scale object detection. RatioNet equipped with ratio-center in single-scale testing achieves 46.4% AP on the test-dev set. It outperforms the best one-stage and key-point detector, CenterNet [16], by 1.5% (46.4% vs. 44.9%) in single-scale testing, and is competitive with two-stage detectors.

The contributions of this paper are summarized as follows:

– We propose a simple and effective approach-RatioNet for object detection. A novel regression method is employed by predicting width, height, l_ratio and t_ratio with global features for more correct bounding boxes.
– A concise CNN structure named ratio-center is presented to preserve the high-quality boxes, and it further facilitates the performance of RatioNet.
– The experimental analysis on MS-COCO demonstrates that RatioNet shows competitive performance (49.7% AP) with the state-of-the-art detectors.

## 2. Related Work

Object detection concentrates on two tasks, classification and regression. With the application of deep learning, the detectors can be split into two types, anchor-based detectors and anchor-free detectors.

Anchor-based Detectors: In anchor-based detectors, anchor boxes are placed densely in feature maps to be classified and regressed as priors for region proposal. The Faster region-based convolutional neural network (Faster R-CNN) [21] introduces region proposal network (RPN), where regions of interest (RoIs) can be generated with anchor boxes. Then these RoIs will be further refined to specific categories. Mask R-CNN [22] can explore masks besides the bounding boxes by appending a mask prediction branch to Faster R-CNN [21]. Cascade R-CNN [23] increases IoU threshold to train a sequence of detectors for the problem of overfitting and mismatch. YOLOv2 [13] and YOLOv3 [14] improves the recall rate and performance with anchor boxes compared to YOLOv1 [24]. SSD [15] adopts variant numbers of anchor boxes to multi-scale feature levels for competitive accuracy. RetinaNet [25] applies denser anchor boxes with focal loss. However, anchor boxes are involved in extensive hyper-parameters, e.g., scales, aspect ratios and numbers, which must be designed manually and carefully.

Anchor-free Detectors: Anchor-free detectors avoid complex requirement of anchor boxes. DenseBox [26] and UnitBox [27] exploit the direction region proposal as prior anchor-free detectors, which have been used in text detection [28] and pedestrian detection [29]. RPDet [9] is a new fine representation of objects as a set of sample points for both localization and recognition. FCOS [19], a fully convolution one-stage object detection, regresses four distances from the point to boundaries to form a box and raises a novel branch of center-ness.

Keypoint-based approaches [16,17,18] generate boxes by predicting particular points and grouping them together. Specifically, CornerNet [17] detects a pair of corner points of a bounding box with the help of corner pooling and embedding vectors to group the corner points together. CenterNet [16] identifies corner points with improved cascaded corner pooling and center points with center pooling. ExtremeNet [18] focuses on four extreme points and one center point of objects. Refs. [16,17,18] all rely on a heavily stacked hourglass network [30] for accurate keypoints detection, resulting in a great performance but also suffering from high FLOPs, memory consumption, long training and testing time.

Different from keypoint-based approaches [16,17,18], with viewpoints in a narrow region, e.g., corner region as positive samples, the proposed RatioNet regards all points in ground-truth boxes as positive samples. In addition, RatioNet predicts the width, height, l_ratio and t_ratio of the box and adds a ratio-center subnet with a different formula and better performance compared to center-ness [19].

## 3. The Proposed Approach

In this section, we first introduce the specifics of RatioNet architecture in Section 3.1. Then the novel regression model consisting of w-h regression and ratio regression is given in Section 3.2. Finally, Section 3.3 presents ratio-center to illustrate how to capture the high-quality locations for optimal prediction.

### 3.1. Overview of the Network Architecture

**Figure 1:**
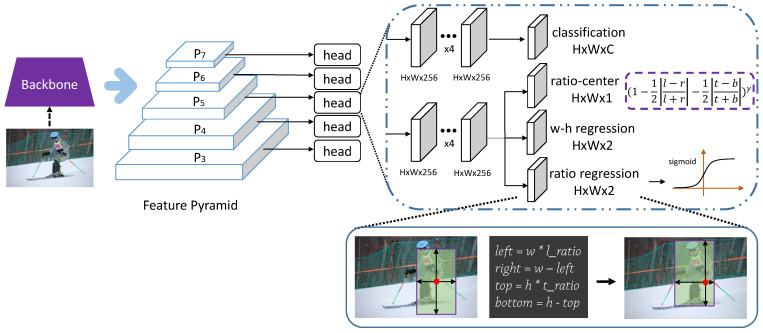
The network architecture of RatioNet. Ratio regression and w-h regression are responsible for predicting bounding boxes. Ratio-center is used to refine the high-quality boxes. Classification is used to predict scores of categories.

The overview of the network architecture is shown as Figure 1. RatioNet is an anchor-free detector constructed with the backbone network and two task-specific subnets, classification and regression.

In the proposed architecture, a feature pyramid network is applied for optimal detection of multi-scale objects, especially the small ones, with features from different levels. Following [19], five levels from P${}_{3}$ to P${}_{7}$ is used, where *l* is defined as the pyramid level. At the same time, P${}_{l}$ also means that the resolution of the feature map in this level is 1/2${}^{l}$ of the input image, that is to say, the strides of 8, 16, 32, 64 and 128 are assigned to the levels of P${}_{3}$, P${}_{4}$, P${}_{5}$, P${}_{6}$, P${}_{7}$, respectively. To be specific, when *l* is in {3, 4, 5}, P${}_{l}$ is generated from the backbone network’s feature maps called C${}_{l}$, which is followed by a 1 × 1 convolutional layer. Moreover, P${}_{6}$ is produced from P${}_{5}$ rather than C${}_{5}$ through a convolutional layer with a stride of 2, and then P${}_{7}$ is produced from P${}_{6}$ through the similar operation. As in [19], experiment have been conducted with higher AP and less memory cost with the application of P${}_{5}$ compared to C${}_{5}$ to generate the feature level of P${}_{6}$. Each level of the feature pyramid network handles different objects with corresponding scales directly, instead of utilizing anchors with different areas in anchor-based detectors.

As shown in Figure 1, every feature level is followed by a detection head. Each head has two fundamental branches, classification and regression. Suppose the size of input image is (W${}_{i}$, H${}_{i}$), and the bounding box of the object in the input image is (*x*${}_{b}$, *y*${}_{b}$, *w*${}_{b}$, *h*${}_{b}$, *c*${}_{b}$), where (*x*${}_{b}$, *y*${}_{b}$), (*w*${}_{b}$, *h*${}_{b}$), *c*${}_{b}$ denote the center coordinate, width and height, and the category of the box, respectively. In addition, (*x*${}_{b}^{l}$, *y*${}_{b}^{l}$, *w*${}_{b}^{l}$, *h*${}_{b}^{l}$, *c*${}_{b}^{l}$) is defined as the relative ground-truth box in the P${}_{l}$, and it is equal to (*x*${}_{b}$/2${}^{l}$, *y*${}_{b}$/2${}^{l}$, *w*${}_{b}$/2${}^{l}$, *h*${}_{b}$/2${}^{l}$, *c*${}_{b}$). Each branch following the detection head is firstly equipped with four 3 × 3 convolutional layers, where the resolution of the input feature map is the same as the output feature map, to further extract deep features.

Classification Subnet: As aforementioned, one of the subnets attached to each feature level is the classification subnet. The shape of the classification output for the *l*th level is (W${}_{c}$, H${}_{c}$, C), where (W${}_{c}$, H${}_{c}$) equal to (W${}_{i}$/2${}^{l}$, H${}_{i}$/2${}^{l}$) denotes the size of each feature map and C denotes the channels of feature maps as well as the total number of classes, which is 80 for COCO dataset [22]. Each spatial location of the classification output predicts the probability of belonging to specific category of objects. We regard all the spatial locations falling into the ground-truth box region, i.e., (*x*${}_{b}^{l}$, *y*${}_{b}^{l}$, *w*${}_{b}^{l}$, *h*${}_{b}^{l}$) in the classification output of P${}_{l}$, as the positive samples, so as many positive samples as possible are utilized in training. To some extent, it alleviates the quantitative imbalance of positive and negative samples, and achieves easier training and better performance compared to the keypoint-based approach. Let *P_ijc_* be the predicted score at the location (*i*, *j*) for class c, and let *Y_ijc_* be the corresponding ground-truth. Following focal loss [25], the classification loss is defined as:

(1) $$L_{cls}=\frac{-1}{N}\sum_{i=1}^{H}\sum_{j=1}^{W}\sum_{c=1}^{C}\left\{\begin{array}{cc} \theta {\left(1-P_{ijc}\right)}^{\eta }\log\left(P_{ijc}\right), & \mathrm{if}\;Y_{ijc}\;=\;1\\ \left(1-\theta \right)P_{ijc}^{\eta }\log\left(1-P_{ijc}\right), & \mathrm{otherwise} \end{array}\right.$$

where θ and η are hyper-parameters equal to 0.25 and 2, respectively, and *N* is the number of positive samples.

Regression Subnet: The other subnet attached to each feature level is the regression subnet. The final layers are mainly the w-h regression and the ratio regression, which are in parallel with each other. The shape of either w-h regression or ratio regression is (W${}_{i}$/2${}^{l}$, H${}_{i}$/2${}^{l}$, 2) for the *l*-th level of the feature pyramid network. We utilize the w-h regression, merged with the ratio-regression, to generate the bounding box, which is introduced in detail in Section 3.2. Moreover, there is another output branch, in parallel with the w-h regression and ratio regression, named ratio-center with the shape of (W${}_{i}$/2${}^{l}$, H${}_{i}$/2${}^{l}$, 1). With the help of ratio-center, a large amount of low-quality bounding boxes are suppressed so that the high-quality bounding boxes get high scores for better performance.

### 3.2. W-H Regression and Ratio Regression

In general, detectors [19,27,31], the regression branch predicts distances from the current location in the corresponding feature maps to the top, bottom, left and right sides of the bounding box. In experiments, this regression pattern primarily considers single oriented features of objects in each channel, that is to say, during inference it’s easy to form false boxes due to the neglect of global features of the object. For better utilization of the overall features, we adopt two branches, w-h regression and ratio regression, to predict the width and height of the boxes and the relative ratios to determine the final boxes.

**Figure 2:**
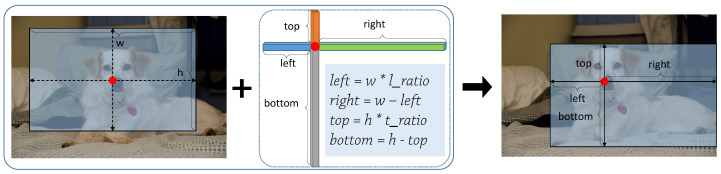
The process of generating a bounding box, where top, bottom, left and right represent the distance from the point to four boundaries, w and h are defined as the width and height of the box, l_ratio and t_ratio mean the ratio of left to width and top to height.

Particularly, the branches of w-h regression and ratio regression are attached to the four 3 × 3 convolutional layers with the shape of (W${}_{i}$/2${}^{l}$, H${}_{i}$/2${}^{l}$, 2). For w-h regression, the pair of values of each pixel location in the feature maps with two channels indicate the width and height of the corresponding box. On the other hand, due to the uncertain position of the point, which falls into the bounding box, the predicted target still cannot be determined. So we need one more constraint, i.e., ratio regression. It is worth noting that ratio regression has the same shape as w-h regression, especially the same number of channels. Firstly, we define l_ratio as the ratio of the distance (from the point in the box to the left boundary) to the width of the box. Similarly, t_ratio is defined as the ratio of the distance (from the point to the top boundary) to the height of the box. Specifically, l_ratio and t_ratio represent the two-channel feature maps of ratio regression. Hence, as shown in Figure 2, if the location (x, y) is associated with a bounding box, the formula of the regression target is defined as,

(2) $$\left\{\begin{array}{c} left=w*l\_ratio,\\ right=w-left,\\ top=h*t\_ratio,\\ bottom=h-top \end{array}\right.$$

With the above four values and the location (x, y), a certain bounding box can be determined. What is more, we add a sigmoid function attached to the ratio-regression to guarantee that the values of l_ratio and t_ratio are in [0, 1]. During training, we apply IoU loss [27] generally used in box regression at the positive locations,

(3) $$L_{reg}=\frac{1}{N}\sum_{i=1}^{H}\sum_{j=1}^{W}-log\left(IoU(P_{ij},Y_{ij}\right),$$

where N is the number of positive samples and *P_ij_* and *Y_ij_* are the predicted box and the ground-truth, respectively.

**Figure 3:**
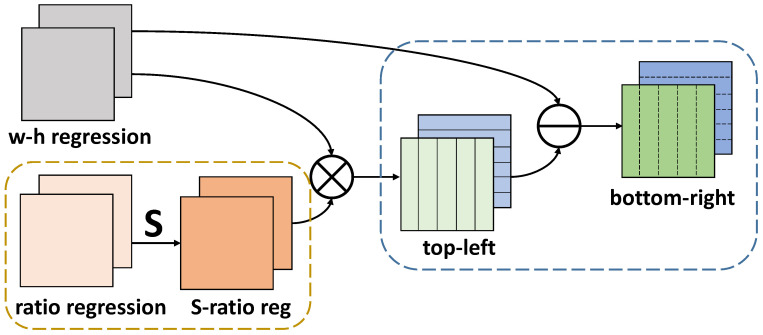
The structure of the cooperation of w-h regression and ratio regression. S denotes the function of sigmoid. S-ratio reg denotes that ratio regression is processed through sigmoid. Top-left and bottom-right mean the feature maps representing distances from the location to top, left, bottom and right boundaries, repectively.

Figure 3 provides the structure of how w-h regression and ratio regression work jointly. Firstly, the ratio regression is processed by the sigmoid function. Then the output feature maps S-ratio reg with two channels are multiplied by w-h regression correspondingly. Finally, w-h regression subtracts top-left to generate the bottom-right with two channels. The total four channels of top-left and bottom-right represent the distances from the current location to the top, left, bottom and right boundaries of the box, respectively.

### 3.3. Ratio-Center

In general, visual regions close to the center region have higher-quality predictions than those far from the center. So ratio-center is proposed to leverage the locations close to the center region as far as possible. The ratio-center is defined as,

(4) $$ratiocenter={\left(1-0.5\left|\frac{l-r}{l+r}\right|-0.5\left|\frac{t-b}{t+b}\right|\right)}^{\gamma },$$

where *l*, *r*, *t*, *b* indicate the distances from the location to four boundaries, γ is the hyper-parameter, and the value of ratio-center is limited in [0, 1]. As we can see, when the location is closer to the center, the value of ratio-center is approaching to 1. So we can employ ratio-center as the weight of each location. The loss of the ratio-center is binary cross entropy (BCE) loss defined as,

(5) $$L_{cen}=-\frac{1}{N}\sum_{i=1}^{H}\sum_{j=1}^{W}\left(Y_{ij}*log\left(P_{ij}\right)+\left(1-Y_{ij}\right)*log\left(1-P_{ij}\right)\right),$$

where N is the number of locations and *P_ij_* and *Y_ij_* are the prediction of the ratio-center and the ground-truth, respectively.

**Figure 4:**
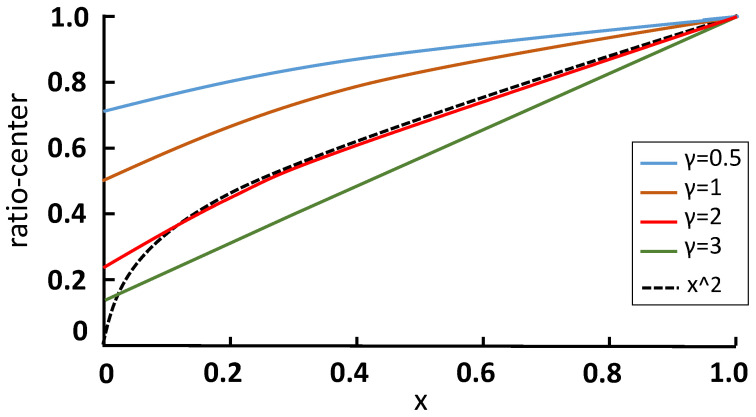
The visualization of Equation (5). We show the curve of Equation (5) when γ = 0.5, 1, 2, 3 as x changes from 0 to 1.0. When x increases, the distance from the current location to the center reduces. The line of dashes represents center-ness [19]. The vertical axis denotes the value of ratio-center or center-ness, meaning the weight of the pixel locations.

In order to show the effect of ratio-center mathematically, we draw the function relation curve with different γ, as shown in Figure 4, by assuming that *t* is equal to *b* and x is defined as the proportion of *l* to *r*. So ratio-center is changed to,

(6) $$ratiocenter={\left(1-0.5\left|\frac{x-1}{x+1}\right|\right)}^{\gamma }.$$

To illustrate the difference with center-ness [19], we also change the center-ness into x${}^{0.5}$ with the same settings as the line of dashes shown in Figure 4. When γ is changed from 0.5 to 3, the gradient of the curve increases. One major difference between ratio-center and center-ness [19] is that when x is 0, center-ness is 0 while ratio-center is larger than 0. Thus, we argue that ratio-center pays softer attention to the location in the marginal region than center-ness. In Section 4.2, we show that the performance of our ratio-center is better than center-ness.

### 3.4. Description

In order to understand the algorithm clearly, we provide an overview of the algorithm in pseudocode, as shown in Algorithm 1. Firstly, for each level of the feature pyramid, we assign the ground-truth to the relative level and then the positive samples and negative samples are defined according to the position. The class score is computed by the classification and ratio-center. Further, the left, right, top and bottom are computed by the output of w-h regression and ration regression. Finally, if train, we will get the loss and update the weights. If test, we will choose the box based on the threshold.
**Algorithm 1** An overiew of the algorithm in pseudocode**Input:** 1:***L*** is the number of feature pyramid levels;2:***S*** is the size interval for different feature pyramid levels;3:*t* is the threshold for classification;**Output:**4:**for** each level i∈[1,L] **do**5:    **ground-truth** *g* is assigned to this level if the size of *g* is in *S${}_{i}$*:6:          *positive samples*: the locations in *g*;7:          *negative samples*: the locations beyond *g*;8:    the output *classification* and *ratio-center* multiply corresponding positions:9:          *class score* = *classification* * *ratio-center*;10:    the output *w-h regression* and *ratio regression* (*l_ratio*, *t_ratio*) generate the box *b*:11:          *left* = *w* *l_ratio;    *right* = *w* – *left*;    *top* = h*t_ratio;    *bottom* = *h* – *top*;12:**end for**13:**If train:**14:      compute the IoU loss and classification loss to update the weights of the network;15:**If test:**16:      choose boxes with class score > t for the output.

## 4. Experiment

RatioNet was evaluated on the MS-COCO [12] dataset, which includes over 1.5 million instances for 80 categories. COCO tainval35k split (80 K images in the training set and 35 K images in the validation set) is used for training and minival split (remaining 5 K images in the validation set) is used in the ablation study. On the test-dev set (20 K images), we explore the test result to compare with prior detectors by submitting detection results to the evaluation server. Unless specified, experiments in ablation study all use ResNet-50 [13] as the backbone. For the main experiment results, we report average precision (AP) over all IOU thresholds (0.50∼0.95), AP${}_{50}$ and AP${}_{75}$ at IOU thresholds 0.50 and 0.75, respectively. AP${}_{S}$, AP${}_{M}$ and AP${}_{L}$ represent the average precision of small objects (area < 32${}^{2}$), medium objects (32${}^{2}$ < area < 96${}^{2}$) and large objects (area > 96${}^{2}$).

### 4.1. Training and Testing Details

Training Details: We utilize the backbone networks ResNet [32] and ResNeXt [33] pretrained on ImageNet [34] to train RatioNet. Following [19], the shorter side of the input image is resized to be 800 while the longer side is less or equal to 1333 for training. Stochastic gradient descent (SGD) is used to optimize the full training loss *L*${}_{det}$,

(7) $$\begin{array}{c} L_{det}=L_{cls}+L_{reg}+L_{cen}, \end{array}$$

which is composed of L${}_{cls}$ for classification, *L*${}_{reg}$ for regression and *L*${}_{cen}$ for ratio-center. *L*${}_{cls}$ and *L*${}_{reg}$ have been defined in Equations (1) and (3), respectively, and *L*${}_{cen}$ is the BCE loss. We train the RatioNet on 2 GTX 1080Ti GPUs with a batch size of 8. We set the maximum number of iterations as 200 K and the initial learning rate as 5 × 10${}^{-3}$. The learning rate is dropped by a factor of 10 at iteration 120 K and 160 K, respectively. Weight decay is 0.0001 and momentum is 0.9. What’s more, we warm up the training through multiplying the initial learning rate by a factor of 1.0/3 during the first 500 iterations, for more stable network learning at the beginning.

Inference Details: After forwarding the input image, the network produces w-h regression and ratio-regression. We utilize the Equation (2) to get the bounding box by relatively multiplying w-h regression by ratio-regression. At the same time, the network produces the classification scores and the ratio-center. We multiply them and then assign the results to the corresponding bounding boxes as the final confidence scores. Then non-maximum suppression is used to exclude the redundant boxes with an IoU threshold of 0.6. Following [31], the size of input images are the same as in training. Unlike [16,17], we don’t adopt flipping or multi-scale testing in inference while we still get the competitive result.

### 4.2. Effect of Ratio-Center

As stated in Section 3.2, RatioNet determines the size of boxes with the use of Equation (2), and the location of boxes with the position of pixels and the corresponding ratios. However, we find that the position of the pixel is essential to the quality of boxes. Ref. [19] by Tian, Z. et al. has demonstrated that locations far from the center of objects tend to produce low-quality boxes. Thus, ref. [19] by Tian, Z. et al. proposes center-ness to suppress the low-quality locations. In this section, we further analyze two causes of low-quality boxes: (1) the locations far from the center usually fall into the quadrate bounding boxes but not the objects (i.e., the background in the box), (2) the locations in the overlap of objects especially in the same category easily regress false boxes due to the influence of the other object.

**Figure 5:**
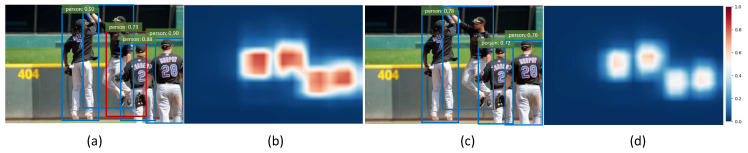
The effect of ratio-center. (**a**,**b**) represent the false bounding box (red) and the corresponding heatmap without ratio-center, respectively. (**c**,**d**) represent the result and the heatmap with ratio-center, respectively, which shows the better performance. The category thresholds are ensured by the F1-score. Only the result of the person category is visualized.

**Figure 6:**
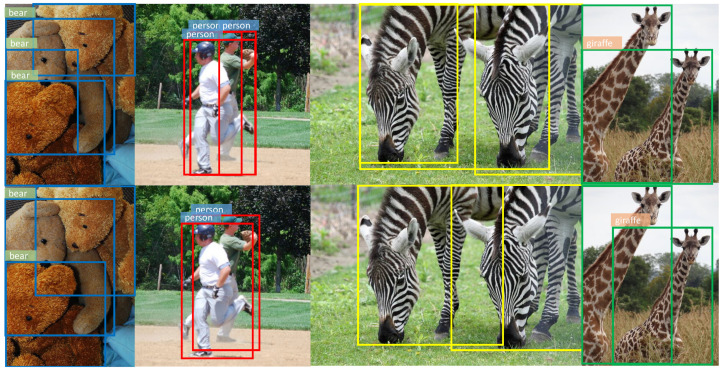
Some compared detection examples with or without ratio-center. There are overlaps between objects in the figures. The first row represents the results without ratio-center, where some false bounding boxes are generated due to the overlap. The second row represents the results with ratio-center, and the false boxes are reduced.

Firstly, we set the category thresholds that result in the maximum F1-score which considers both the precision and recall. As shown in Figure 5a, without ratio-center, we get a false box (red) which covers the overlap of the two persons. In Figure 5b, the category heatmap of (a) is visualized, indicating that the locations in the overlap still get relatively high scores, which leads to the false box covering both the objects due to the similar features of instances in the same category. In Figure 5c, we make use of ratio-center and it can be seen that all predicted boxes are right and the confidence scores are reduced as well as the category thresholds. In Figure 5d, the heatmap of (c) illustrates that the scores in the overlap reduce significantly and obvious dividing lines appear between different objects. We also find that scores in the center region of objects has a certain degree of decline as well, but the result is indeed improved as shown in Figure 5c and Table 1. It turns out that locations in the overlap, i.e., far away from the center obtain lower weights than those close to the center region because of ratio-center. More examples are shown in Figure 6. Additionally, it can be found that the high scores in the heatmap are mainly grouped in the center region of each object, as shown in Figure 5b,d, representing locations in the marginal region obtain low scores even without ratio-center and eliminating the situation that locations fall into the background in the bounding box to some extent. Therefore, it’s not necessary to assign the weights of nearly 0 to the marginal pixels, in other words, we can pay softer attention to the marginal pixels with ratio-center compared to center-ness [19], as shown in Figure 4.

As for the hyper-parameter γ in Equation (4), Table 1 demonstrates that γ = 2 leads to the best performance and outperforms center-ness [19]. The lowest AP gets 35.9% when γ = 0 meaning no use of ratio-center. When γ = 2, the result 37.5% improves 1.6% and 0.4% over APs in γ = 0 and center-ness, respectively. So γ = 2 is for all the following experiments.

### 4.3. Ablation Study

#### 4.3.1. Regression Loss

The use of regression loss in object detectors is mainly divided into Smooth L1 loss [8,10] and IoU loss [19,27]. In this section, we explore the effect of Smooth L1 loss and IoU loss for RatioNet by experiments. Firstly, we apply Smooth L1 loss to w-h regression and ratio regression directly, and add them together as the regression loss. During training, we find that the values of regression loss and classification loss have a large gap, which causes almost no decline of classification loss and bad performance (27.1% AP). Therefore, the weights defined as ϵ and σ are assigned to regression loss and classification loss, respectively. As shown in Table 2, we would like to increase the weight of the classification loss, and as the same time, decrease the weight of the regression loss. Firstly, when ϵ is 0.2 and σ is 2.5, the result becomes better by a large margin. We further change ϵ and σ, and the best result is 35.9% AP when ϵ = 0.25 and σ = 3.0. However, there is still a performance gap 1.2% in AP with [19]. We argue that Smooth L1 loss is so sensitive to the absolute size of the bounding box that there is an imbalance between small and big objects. Thus, we adopt IoU loss [27] as the regression loss with the result 37.5% AP as shown in Table 2. It has an increment of 1.6% compared to Smooth L1 loss. So, IoU loss is applied to the following experiments.

#### 4.3.2. Comparison with Anchor-Based Detectors

We compare our anchor-free detector RatioNet with anchor-based detectors RetinaNet [25] and FPN [35], as shown in Table 3. Following [19], RatioNet makes use of P${}_{5}$ to generate P${}_{6}$ and P${}_{7}$ rather than C${}_{5}$, and other hyper-parameters, like the NMS threshold, are the same as RetinaNet [25]. When ResNet-50 [32] is used as the backbone network, on the MS COCO dataset [2], RatioNet outperforms RetinaNet [25] and FPN-RoIAlign (i.e., Faster R-CNN equipped with Feature Pyramid Networks.) [35] by 1.8% AP and 0.8% AP, respectively, likely because of the novel box regression and the utilization of ratio-center for RatioNet. We use RetinaNet, FPN-RoIAlign and our RatioNet for the comparable experiment on the VOC2012 dataset [11]. The results show that RetinaNet and FPN-RoIAlign get the 75.1% AP and 74.9% AP, while our RatioNet gets the 78.1% AP. The parameters of RetinaNet, FPN-RoIAlign and our RatioNet are 37.96 M, 41.75 M and 32.24 M. That is to say, the computational cost of RatioNet is less than RetinaNet and FPN-RoIAlign. RatioNet is a simple and effective approach. This further indicates that the performance of anchor-free detectors has the potential to exceed the anchor-based detectors.

More importantly, RatioNet, an anchor-free detector, avoids hyperparameters such as the aspect ratio and scales of anchors, which must be designed carefully in anchor-based detectors. It also illustrates that though anchor can help to regress the box, it’s not necessary for object detection and we can get a better result without an anchor. We believe that the research of anchor-free detectors will be a popular stream in the future object detection field.

#### 4.3.3. Performance of Each Component

We explore the contribution of components, i.e., w-h regression, ratio-regression and ratio-center, by comparing with the baseline FCOS [19]. Specifically, in FCOS [19], we define the operation of predicting distances from pixel locations to left, right, top and bottom boundaries as l-r-t-b regression. The w-h-ratio regression represents the operation of w-h regression and ratio regression in RatioNet. In principle, l-r-t-b regression and center-ness in FCOS [19] can be correspondingly replaced by w-h-ratio regression and ratio-center. So we do experiments with different combination modes, such as l-r-t-b regression and ratio-center, to confirm the proposed methods are effective.

As shown in Table 4, FCOS [19] is composed of l-r-t-b regression and center-ness with the result of 37.1% AP. When the network replaces center-ness with ratio-center or replaces l-r-t-b regression with w-h-ratio regression separately, we can observe 0.1% or 0.2% AP improvements over the baseline FCOS [19], respectively. This indicates that either w-h-ratio regression or ratio-center benefits the performance of the network. The proposed RatioNet is equipped with both the w-h-ratio regression and ratio-center, which results in 37.5% AP and promotes the performance by 0.4% AP. The result suggests that the combination of w-h-ratio regression and ratio-center benefits the detection capability of the network. For equal comparison, we keep the hyper-parameters and initialization between FCOS [19] and RatioNet unchanged, except w-h-ratio regression and ratio-center.

#### 4.3.4. Results of Different Levels of Feature Pyramid Networks

The parameters of the proposed model with all the {P${}_{3}$, P${}_{4}$, P${}_{5}$, P${}_{6}$, P${}_{7}$} levels are 3.86M. We do the experiments with the levels of C${}_{5}$, {P${}_{3}$, P${}_{4}$, P${}_{5}$}, {P${}_{3}$, P${}_{4}$, P${}_{5}$, P${}_{6}$} and {P${}_{3}$, P${}_{4}$, P${}_{5}$, P${}_{6}$, P${}_{7}$} respectively to illustrate how the number of levels of the feature pyramid network affects the performance. As shown in the Table 5, the result with all the {P${}_{3}$, P${}_{4}$, P${}_{5}$, P${}_{6}$, P${}_{7}$} levels is the best one (37.5% AP) with limited computation increment (+0.71 M). So we utilize all the five levels in our proposed RatioNet.

### 4.4. State-of-the-Art Comparisons

The object detection methods are mainly the two-stage detectors and one-stage detectors. Faster R-CNN [21] and Cascade R-CNN [23] are representative in two-stage detectors with the performance of 36.2% AP and 42.8% AP with the backbone of ResNet-101. Our RatioNet has the comparative result 42.7% AP as shown in the Table 6. In one-stage detectors, RetinaNet [25] and FSAF [31] can achieve the result 39.1% AP and 42.9% AP with the backbone of ResNet-101 and ResNeXt-101, while our RatioNet can get the performance of 43.8% AP with the backbone of ResNeXt-101. The keypoint-based and one-stage detectors, CornerNet [13], ExtremeNet [18] and CenterNet [16] get the results of 40.6% AP, 40.2% AP and 44.9% AP, respectively, with the backbone of HourglassNet-104 that is only utilized in the keypoint-based detectors but not in other detectors. The proposed RatioNet can achieve 46.4% AP with the backbone of ResNeXt-101-DCN, which is better than all the keypoint-based detectors. RatioNet is better than the real-time detectors such as YOLO [13] and SSD [15] in AP, though its speed is lower than the latter.Because of the use of multiple levels of feature pyramid network, the computation of RatioNet increases as well as the time of testing an image. The size of the input image in YOLO is 416 × 416 while the size of the input image in RatioNet is resized to be 800 while the longer side is less or equal to 1333. So RatioNet is slower than YOLO. The time of training RatioNet with a batch size of 8 and an iteration of 200 K is about 23 h, while the time of testing an image is 48 ms on a GTX 1080Ti GPU. RatioNet is competitive to previous state-of-the-art one-stage and two-stage detectors. It is worth mentioning that RatioNet gets the best result 49.7%AP with multi-scale testing as shown in the last row of Table 6.

## 5. Conclusions

This paper proposes a simple and effective anchor-free detector RatioNet, and presents a novel regression method based on the relative ratio prediction and width and height of the box for object detection. Then we can generate the boxes with these clues, i.e., ratio, width and height. This method lies in that it leverages the global features of objects for accurate bounding box prediction. As for the architecture of the model, we utilize the feature pyramid network for various sizes of objects by integrating the features of the high semantic levels and low levels, which also improves the detection results of the model. What’s more, we propose ratio-center for better utilization of the high-quality pixel locations (the central region of the box) which promotes the performance by a large margin (+3.4% AP). Ablation study shows that the proposed ratio-center is more effective than centerness in FCOS. The proposed RatioNet has fewer parameters than other detectors such as RetinaNet. Extensive experiments illustrate that the proposed RatioNet can achieve comparable performance (49.7% AP) with state-of-the-art detectors.

The ratio-center distributes high weight to the central region and gets great gains. In future research, we will major in the attention mechanism to learn the weight of each location, which means not only the central region but also other vital locations with significant features can be highly weighted.

## Figures and Tables

**Table 1:** The results for different γ of ratio-center in RatioNet. When γ = 2, RatioNet gets 37.5% AP, which outperforms FCOS [19] with center-ness 0.4% AP. The bold means the best performance.

Method	γ	AP	AP${}_{50}$	AP${}_{75}$	AP${}_{S}$	AP${}_{M}$	AP${}_{L}$
center-ness [31]	-	37.1	55.9	39.8	21.3	41.0	47.8
ratio-center	0	35.9	51.2	37.5	19.2	38.4	44.3
	1	36.7	54.7	38.7	20.9	40.1	46.1
	2	**37.5**	56.8	39.9	22.0	41.5	48.0
	3	36.4	54.6	37.9	20.1	39.8	46.4

**Table 2:** The results for different settings for smooth L1 loss and intersection over union (IoU) loss.

Method	ϵ	σ	AP	AP${}_{50}$	AP${}_{75}$	AP${}_{S}$	AP${}_{M}$	AP${}_{L}$
Smooth L1 loss	1	1	27.1	48.0	23.5	8.9	30.5	41.5
	0.2	2.5	34.0	54.1	36.3	16.4	36.7	43.4
	0.25	2.5	35.1	54.6	36.9	17.1	37.1	45.9
	0.25	3.0	35.9	55.0	38.1	17.8	38.0	44.7
IoU loss	-	-	**37.5**	56.8	39.9	22.0	41.5	48.0

**Table 3:** Comparison of the proposed RatioNet and the anchor-based approaches on the dataset of VOC2012 [11] and MS COCO [2].

Method	Backbone	Anchors per Scale	Parameters	AP#COCO	AP#VOC
RetinaNet [21]	ResNet-50	3 × 3	37.96 M	35.7	75.1
FPN-RoIAlign [20]	ResNet-50	3 × 1	41.75 M	36.7	74.9
RatioNet(ours)	ResNet-50	-	32.24 M	**37.5**	**78.1**

**Table 4:** The results of w-h-ratio regression and ratio-center. l-r-t-b regression denotes the method of predicting the distances from locations to left, right, top and bottom boundaries. w-h-ratio regression denotes the method of w-h regression and ratio regression in our approach.

Method	l-r-t-b Regression	Center-ness	w-h-Ratio Regression	Ratio-Center	AP
FCOS [31]	*√*	*√*			37.1
	*√*			*√*	37.2
		*√*	*√*		37.3
ours			*√*	*√*	**37.5**

**Table 5:** The parameters and results of the feature pyramid network in different levels in the proposed RatioNet.

Levels of FPN	Parameters	AP	AP${}_{50}$
C5	–	31.8	51.2
{P${}_{3}$, P${}_{4}$, P${}_{5}$}	2.71 M	35.1	53.4
{P${}_{3}$, P${}_{4}$, P${}_{5}$, P${}_{6}$}	3.15 M	36.0	54.3
{P${}_{3}$, P${}_{4}$, P${}_{5}$, P${}_{6}$, P${}_{7}$}	3.86 M	**37.5**	**56.8**

**Table 6:** Comparisons with sate of the art on COCO test-dev set with single-scale tesing for all methods. R: ResNet. X: ResNeXt. HG: Hourglass Network. DCN: Deformable Convolutional Network [36,37]. Our best model with X-32x8d -101-DCN outperforms the best one-stage detector CenterNet by 1.5% AP. It is comparable with two-stage detectors. * means multi-scale testing.

Method	Backbone	AP	AP${}_{50}$	AP${}_{75}$	AP${}_{S}$	AP${}_{M}$	AP${}_{L}$
**two-stage detectors:**							
Faster R-CNN w/FPN [35]	R-101	36.2	59.1	39.0	18.2	39.0	48.2
Cascade R-CNN [23]	R-101	42.8	62.1	46.3	23.7	45.5	55.2
Libra R-CNN [38]	X-101	43.0	64.0	47.0	25.3	45.6	54.6
RPDet [9]	R-101	41.0	62.9	44.3	23.6	44.1	51.7
RPDet [9]	R-101-DCN	42.8	65.0	46.3	24.9	46.2	54.7
TridenNet [39]	R-101	42.7	63.6	46.5	23.9	46.6	56.6
TridenNet [39]	R-101-DCN	**46.8**	67.6	51.5	28.0	51.2	60.5
**one-stage detectors:**							
YOLOv2 [13]	DarkNet-19	21.6	44.0	19.2	5.0	22.4	35.5
SSD513 [15]	R-101	31.2	50.4	33.3	10.2	34.5	49.8
DSSD513 [7]	R-101	33.2	53.3	35.2	13.0	35.4	51.1
RetinaNet [25]	R-101	39.1	59.1	42.3	21.8	42.7	50.2
CornerNet [17]	HG-104	40.6	56.4	43.2	19.1	42.8	54.3
ExtremeNet [18]	HG-104	40.2	55.5	43.2	20.4	43.2	53.1
CenterNet [16]	HG-104	44.9	62.4	48.1	25.6	47.4	57.4
FSAF [31]	X-101	42.9	63.8	46.3	26.6	46.2	52.7
FCOS [19]	X-101	43.2	62.8	46.6	26.5	46.2	53.3
RatioNet(ours)	R-101	42.7	61.8	45.9	25.0	45.8	54.5
RatioNet(ours)	X-101	43.8	62.6	47.9	25.6	46.4	55.9
RatioNet(ours)	R-50-DCN	42.4	61.4	46.0	24.2	45.2	54.5
RatioNet(ours)	R-101-DCN	44.4	63.5	48.5	26.3	47.3	56.7
RatioNet(ours)	X-101-DCN	**46.4**	65.8	50.6	28.4	49.4	59.2
RatioNet*(ours)	X-101-DCN	**49.7**	67.1	51.3	29.6	50.1	60.1

## Data Availability

Data available in a publicly accessible repository that does not issue DOIs. Publicly available datasets were analyzed in this study. This data can be found here: https://cocodataset.org/ (accessed on 26 February 2021).
